# Phenotypic Variability and Anticancer Alkaloid Profiles of *Catharanthus roseus* Cultivars Grown Under a Vertical Farming System

**DOI:** 10.3390/plants14162576

**Published:** 2025-08-19

**Authors:** Marisa S. C. Lourenço, Victor Freitas, Ep Heuvelink, Susana M. P. Carvalho

**Affiliations:** 1 GreenUPorto—Sustainable Agrifood Production Research Centre/Inov4Agro, DGAOT, Faculty of Sciences, University of Porto, Campus de Vairão, Rua da Agrária 747, 4485-646 Vairão, Portugal; marisa.lourenco@fc.up.pt; 2 REQUIMTE—Faculty of Sciences of University of Porto, Rua do Campo Alegre, s/n, 4169-007 Porto, Portugal; vfreitas@fc.up.pt; 3 Horticulture and Product Physiology Group, Department of Plant Sciences, Wageningen University, 6708 PB Wageningen, The Netherlands; ep.heuvelink@wur.nl

**Keywords:** controlled environment, medicinal plants, terpenoid indole alkaloids, plant molecular factories, vinblastine, vinca, vincristine

## Abstract

Plants are promising biofactories for high-value compounds, and integrating vertical farming (VF) with plant molecular farming (PMF) enhances the efficiency and sustainability of these systems. *Catharanthus roseus* (L.) is the only natural source of vinblastine (VLB) and vincristine (VCR), key anticancer alkaloids used in chemotherapy. This study assessed the morpho-physiological responses and the organ-specific anticancer-related terpenoid indole alkaloid (TIA) production in nine *C. roseus* cultivars grown in a VF system. Results revealed a significant intraspecific variability (between and within plant series) concerning both plant growth and alkaloid profile. Although total anticancer-related TIA concentration was 1.6- to 5.9-fold higher in leaves than in flowers, the key anticancer alkaloids VLB and VCR exhibited distinct patterns depending on the cultivar, with ‘C-Red’ showing a higher concentration of both alkaloids in leaves, while ‘C-XDR-PN’ and ‘C-XDR-WT’ had a significantly higher concentration of VCR in flowers (3.15 and 4.05-times higher, respectively). This cultivar-dependent variability, in the production of specific anticancer alkaloids, highlights the importance of a proper cultivar selection for their commercial production. Our findings show that VCR concentration may serve as a more reliable cultivar selection marker for anticancer alkaloid yield than total biomass or overall TIA content in VF systems.

## 1. Introduction

While plants have long been cultivated for medicinal purposes, plant molecular farming (PMF; i.e., the use of plants to produce complex molecules of high commercial value such as therapeutic agents) continues to evolve through innovations in biotechnology and synthetic biology [1]. Indeed, PMF has unique advantages over other systems, since it represents a more cost-effective, scalable, and environmentally sustainable approach than traditional chemical synthesis or microbial production systems [2,3]. Its potential can be further enhanced by vertical farming (VF), a high-efficiency cultivation system that allows stacked plant growth under LED lighting in fully controlled environments [4]. In recent years, VF systems have experienced rapid growth, but their high capital investment and energy demands [5] underscore the need to focus on cultivating high-value crops to ensure economic viability and sustainability. Since VF enables standardized year-round production and high yields per square meter [6], this makes it well-suited for PMF applications targeting valuable plant-derived molecules. Nonetheless, for optimizing the biosynthesis of these valuable compounds, more studies are required to understand the impact of VF systems on their production and to explore intraspecific variability in the production and partitioning of a target molecule within a given species.

Cancer remains a major global health problem [7,8], and conventional treatments often involve severe side effects, prompting the search for natural alternatives [9,10]. Alkaloids, with their structural complexity, are promising candidates in anticancer drug development [11,12]. *Catharanthus roseus* (L.), an ornamental plant native to Madagascar, is the only known natural source of the anticancer alkaloids vinblastine (VLB) and vincristine (VCR), two highly valuable compounds due to their application in chemotherapy [13]. Although the plant produces over 130 terpenoid indole alkaloids (TIAs) [14], the commercial use of VLB and VCR is constrained by their low natural concentration in plant tissues.

**Figure 1:**
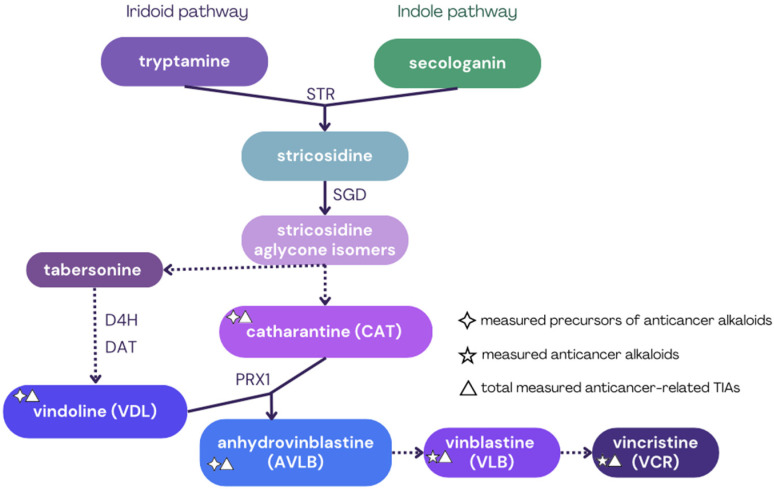
Simplified biosynthetic pathway production of specific terpenoid indole alkaloids (TIAs) in *Catharanthus roseus*, illustrating the two converging pathways: the iridoid pathway (starting from tryptamine) and the indole pathway (starting from secologanin). Solid lines represent direct enzymatic reactions, while dashed lines indicate multistep processes. Abbreviations: D4H: Deacetoxyvindoline 4-Hydroxylase; DAT: Deacetylvindoline 4-O-Acetyltransferase; PRX1: Peroxidase 1; SGD: Strictosidine β-Glucosidase; STR: Strictosidine Synthase. This scheme is a summary of the detailed pathway based on information from studies on *C. roseus* alkaloid biosynthesis [15,16,17,18,31,32]. Symbols in the legend indicate the measured alkaloids in this study.

Anticancer alkaloids are synthesized through a complex multistep biosynthetic pathway [15,16,17,18], briefly illustrated in Figure 1. This pathway is a model for plant specialized metabolism due to its cellular complexity and subcellular compartmentalization [19]. Previous studies have demonstrated that TIAs, including both precursors and anticancer compounds, are predominantly synthesized and accumulated in the leaves of *Catharanthus roseus* [15,20], resulting in higher TIA concentrations in leaves compared to roots or flowers [21]. However, individual alkaloid distribution can vary significantly among organs. For instance, CAT and VDL are commonly detected in leaves, flowers, and shoots, though leaves consistently show the highest levels, often alongside measurable AVLB concentrations [15]. Some studies report low or undetectable AVLB levels, suggesting that the dimerization step may be limiting and influenced by cultivar or growth conditions [22,23,24]. In such cases, the major biochemical bottleneck for anticancer alkaloid biosynthesis appears to be the conversion of AVLB into VLB, a step that remains poorly characterized. Regarding the anticancer alkaloids, VLB is most concentrated in leaves, followed by flowers, seedlings, and stems [21,25,26], while VCR also accumulates primarily in leaves, with only minor amounts found in flowers [21,27,28]. This pattern suggests a strong organ-specific production, with leaves serving as the primary site for commercial extraction of alkaloids due to their higher overall content [29,30].

*Catharanthus roseus* is an economically valuable crop with a short cultivation cycle and compact growth habit, making it well-suited for VF systems. However, limited research has explored the adaptability and phenotypic variability of *C. roseus* cultivars under VF conditions [29]. Magnotta (2006) [14] and Chung (2011) [30] screened 50 and 64 cultivars, respectively, for VDL and CAT content, but neither study quantified the key anticancer alkaloids VLB and VCR. Under controlled chamber conditions (25 °C, 16 h photoperiod), Chung (2011) [30] reported high CAT and VDL levels in ‘Pacifica Peach’ (2.90 and 2.08 mg·g^−1^ DW, respectively). Magnotta (2006) [14], working in a greenhouse, observed CAT ranging from 1.1 to 5.4 mg·g^−1^ FW and VDL from 0.40 to 1.40 mg·g^−1^ FW across most cultivars, with ‘Mediterranean DP Orchid’ notably producing only 0.10 mg·g^−1^ FW of VDL. Expanding such studies to include VLB and VCR under VF conditions may uncover contrasting TIA profiles and help identify important markers for selecting high-yielding genotypes.

This study explores cultivar-specific variation in *C. roseus* growth, physiological performance, and alkaloid production in leaves and flowers under controlled VF conditions. While it is well established that TIAs predominantly accumulate in the leaves, limited research has addressed whether the partitioning and yield of anticancer alkaloids, specifically VLB and VCR, vary according to cultivar and plant organ. Guedes et al. (2024) [15] previously proposed that the limited availability of AVLB represents a key biosynthetic bottleneck in the anticancer alkaloid pathway. However, this assumption has rarely been tested across cultivars grown under controlled conditions. By addressing this gap, this study represents the first comparative analysis of *C. roseus* cultivars under VF targeting VLB and VCR, rather than only their upstream precursors. Additionally, we assess the viability of integrating VF and PMF as a strategy to improve the production of high-value plant-derived compounds. This study investigates whether certain cultivars preferentially accumulate these alkaloids in leaves or flowers, and whether floral tissues could serve as an alternative or complementary extraction source. These insights are critical for optimizing cultivar selection and improving the efficiency of pharmaceutical alkaloid production in controlled environments.

## 2. Results

### 2.1. Morphological and Physiological Parameters

**Figure 2:**
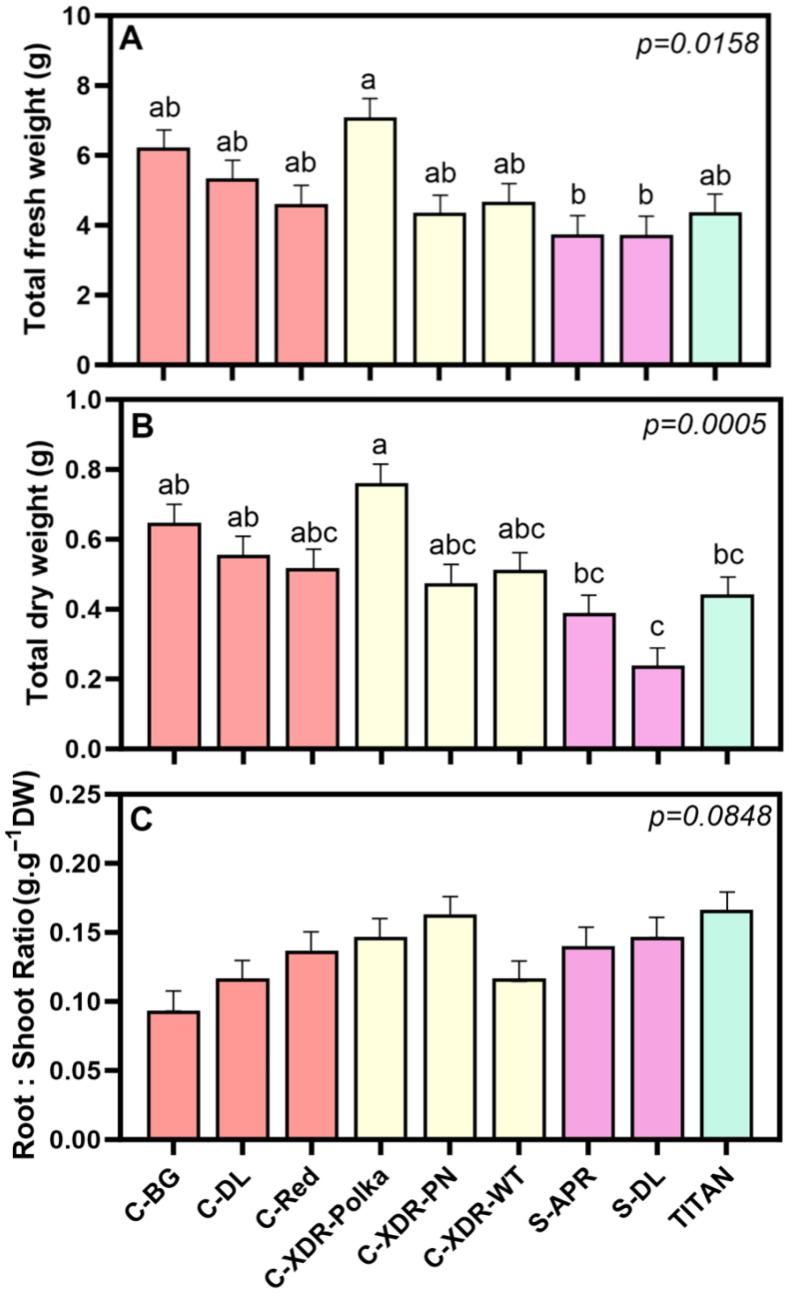
Total fresh weight (**A**), total dry weight (**B**), and root/shoot ratio (**C**) of nine *Catharanthus roseus* cultivars 35 days after transplanting to a vertical farming system. The cultivars tested were Cora Burgundy (C-BG), Cora Deep Lavender (C-DL), Cora Red (C-Red), Cora XDR Polka Dot (C-XDR-Polka), Cora XDR Punch (C-XDR-PN), Cora XDR White (C-XDR-WT), Sunstorm Apricot (S-APR) Sunstorm Deep Lilac (S-DL), and Titan Polka Dot (TITAN). Mean values are based on three independent replicates (n = 3, each composed of five plants). Error bars represent SEM, which is the mean of the standard errors, based on the common variance. *p*-values indicate the f-probability for cultivar differences. Different letters above the bars indicate statistically significant differences between cultivars, as determined by Tukey’s HSD test at *p* = 0.05.

**Figure 3:**
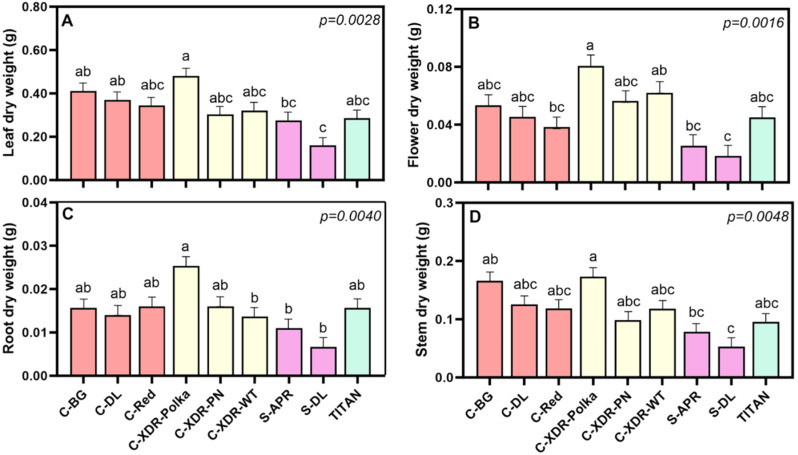
Dry weight partitioning among different plant organs, leaf (**A**), flower (**B**), root (**C**), and stem (**D**), of nine *Catharanthus roseus* cultivars 35 days after transplanting to a vertical farming system. The cultivars tested were Cora Burgundy (C-BG), Cora Deep Lavender (C-DL), Cora Red (C-Red), Cora XDR Polka Dot (C-XDR-Polka), Cora XDR Punch (C-XDR-PN), Cora XDR White (C-XDR-WT), Sunstorm Apricot (S-APR) Sunstorm Deep Lilac (S-DL), and Titan Polka Dot (TITAN). Mean values are based on 3 independent replicates (n = 3; each composed of 5 plants). Error bars represent SEM, which is the mean of the standard errors, based on the common variance. *p*-values indicate the f-probability for cultivar differences. Different letters above the bars indicate statistically significant differences between cultivars, as determined by Tukey’s HSD test at *p* = 0.05.

After 35 days under the established VF conditions, we evaluated the morpho-physiological responses of nine *C. roseus* cultivars. Significant variability in biomass production was observed among cultivars, with ‘Cora-XDR-Polka’ exhibiting a 3.17-fold higher total dry weight (TDW) compared to ‘S-DL’ (Figure 2B). Interestingly, this trend was consistent across all plant organs, with ‘Cora-XDR-Polka’ significantly outperforming the two *Sunstorm* cultivars in shoot, root, and leaf biomass (Figure 3). Despite these differences in biomass production, the root-to-shoot ratio (R:S) did not vary significantly among cultivars (Figure 2C), indicating a relatively uniform dry matter partitioning between above- and below-ground plant tissues.

**Figure 4:**
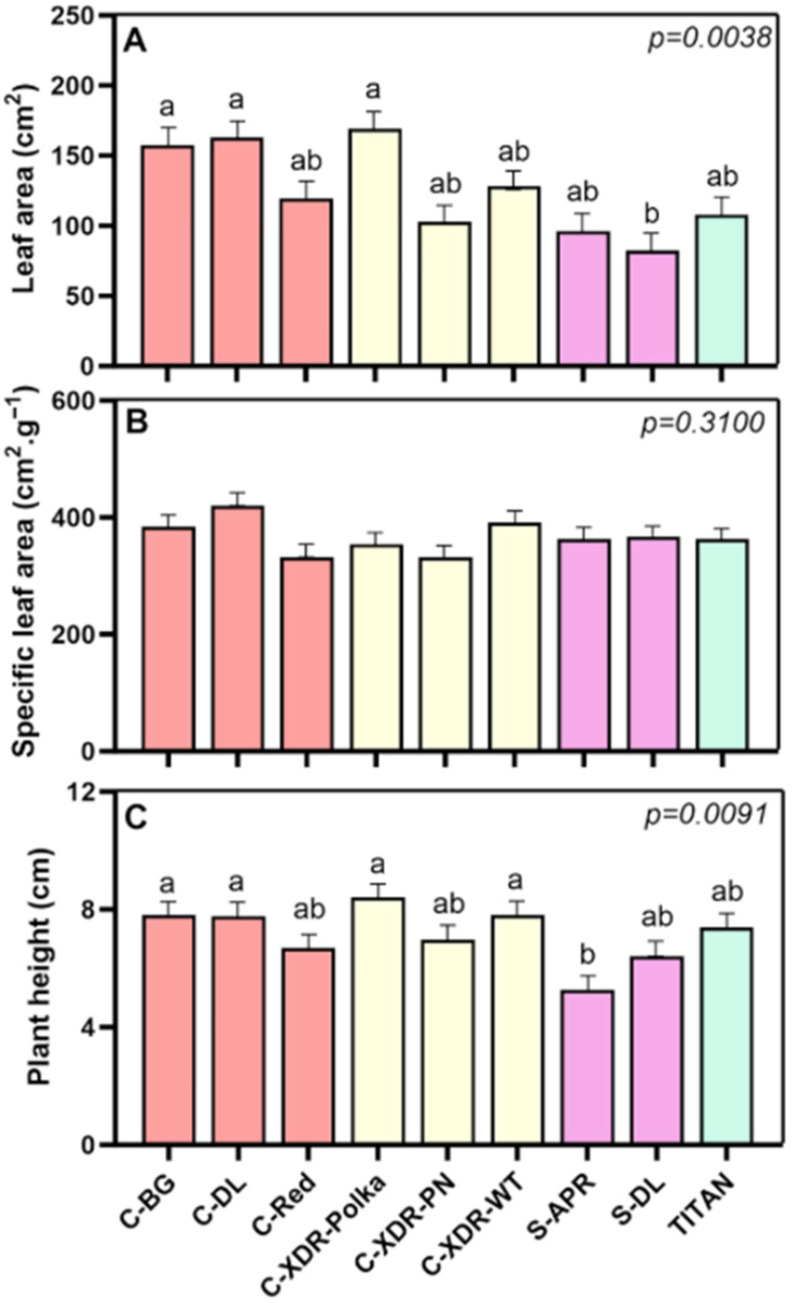
Leaf area (**A**), specific leaf area (**B**), and plant height (**C**) of nine *Catharanthus roseus* cultivars 35 days after transplanting to a vertical farming system. The cultivars tested were Cora Burgundy (C-BG), Cora Deep Lavender (C-DL), Cora Red (C-Red), Cora XDR Polka Dot (C-XDR-Polka), Cora XDR Punch (C-XDR-PN), Cora XDR White (C-XDR-WT), Sunstorm Apricot (S-APR) Sunstorm Deep Lilac (S-DL), and Titan Polka Dot (TITAN). Mean values are based on 3 independent replicates (n = 3; each composed of 5 plants). Error bars represent SEM, which is the mean of the standard errors based on the common variance. *p*-values indicate the f-probability for cultivar differences. Different letters above the bars indicate statistically significant differences between cultivars, as determined by Tukey’s HSD test at *p* = 0.05.

Differences in leaf area (LA) among cultivars were also observed and are aligned with the variations in dry weight. The ‘S-DL’ cultivar exhibited the lowest LA, while ‘C-BG’, ‘C-DL’, and ‘C-XDR-Polka’ showed leaf areas that were up to twice as large (Figure 4A). These differences show a varying capacity for light capture among the cultivars. Despite total LA varied significantly, specific leaf area (SLA)—which reflects leaf thickness and efficiency—remained relatively stable across cultivars (Figure 4B), indicating similar light capture efficiency per unit of leaf mass. Concerning plant height, this trait varied between 6.42 and 8.41 cm for most cultivars, with only ‘S-APR’ showing significantly shorter plants (5.3 cm) (Figure 4C).

**Figure 5:**
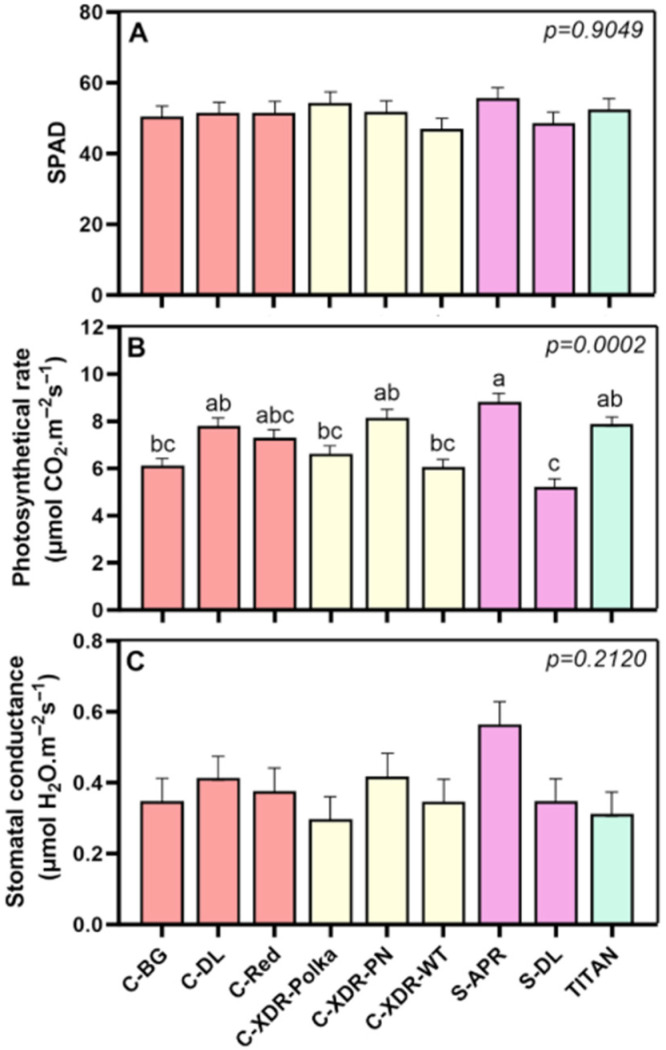
SPAD index (**A**), photosynthetic rate (**B**), and stomatal conductance (**C**) of nine *Catharanthus roseus* cultivars 35 days after transplanting to a vertical farming system. The cultivars tested were Cora Burgundy (C-BG), Cora Deep Lavender (C-DL), Cora Red (C-Red), Cora XDR Polka Dot (C-XDR-Polka), Cora XDR Punch (C-XDR-PN), Cora XDR White (C-XDR-WT), Sunstorm Apricot (S-APR) Sunstorm Deep Lilac (S-DL), and Titan Polka Dot (TITAN). Mean values are based on 3 independent replicates (n = 3, each composed of 5 plants). Error bars represent SEM, which is the mean of the standard errors, based on the common variance. *p*-values indicate the f-probability for cultivar differences. Different letters above the bars indicate statistically significant differences between cultivars, as determined by Tukey’s HSD test at *p* = 0.05.

Despite no significant differences in chlorophyll content being found (SPAD index; Figure 5A), photosynthetic rates varied both within and across series. Notably, among the two *Sunstorm* cultivars, ‘S-APR’ exhibited a 69% higher photosynthetic rate than ‘S-DL’ (Figure 5B), which was accompanied by a higher (though not statistically significant) stomatal conductance (Figure 5C). This may reflect more efficient CO_2_ assimilation in ‘S-APR’, potentially facilitated by greater gas exchange capacity. Interestingly, cultivars such as ‘C-BG’ and ‘C-XDR-Polka’, despite achieving a significantly higher TDW displayed lower photosynthetic rate than ‘S-APR’.

### 2.2. Alkaloid Profile

**Figure 6:**
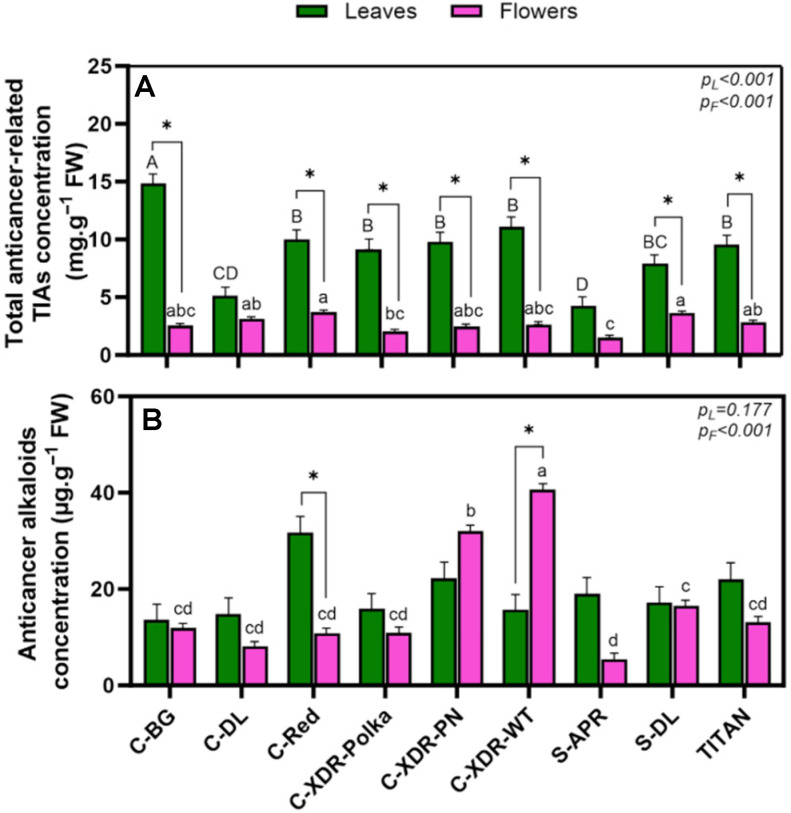
Total anticancer-related TIA concentration [measurements included catharanthine (CAT), vindoline (VDL), anhydrovinblastine (AVLB), vinblastine (VLB), and vincristine (VCR)] (**A**), along with the concentration of specific anticancer alkaloids (vinblastine and vincristine) (**B**) in the leaves and in the flowers of nine *Catharanthus roseus* cultivars 35 days after transplanting to a vertical farming system. The cultivars tested were Cora Burgundy (C-BG), Cora Deep Lavender (C-DL), Cora Red (C-Red), Cora XDR Polka Dot (C-XDR-Polka), Cora XDR Punch (C-XDR-PN), Cora XDR White (C-XDR-WT), Sunstorm Apricot (S-APR) Sunstorm Deep Lilac (S-DL), and Titan Polka Dot (TITAN). Mean values are based on three independent replicates (n = 3), each composed of five plants. Error bars represent the standard error of the mean (SEM), calculated from the common variance. *p*-values indicate the *f*-probability for cultivar differences. Different lowercase and uppercase letters above the bars indicate statistically significant differences between cultivars within each organ – flowers and leaves, respectively – as determined by Tukey’s HSD test (*p* = 0.05). Additionally, a paired *t*-test (df = 2, critical t = 4.303) was performed to assess significant differences between leaves and flowers within each cultivar, with significant differences marked by an asterisk (*).

There was a significant cultivar effect on the concentration of total anticancer-related TIAs, both at leaf and flower levels (Figure 6A). In leaves, the highest concentration was recorded in ‘C-BG’, while ‘C-DL’ and ‘S-APR’ had the lowest values, representing up to a 72% reduction relative to ‘C-BG’. In floral tissues, ‘C-Red’ and ‘S-DL’ showed the highest concentrations, which were significantly greater than those measured in ‘C-XDR-Polka’ and ‘S-APR’. When comparing TIA concentrations between organs, all cultivars except ‘C-DL’ and ‘S-APR’ showed significantly higher levels in leaves, with fold differences ranging from 1.68 (‘S-DL’) to 5.85 (‘C-BG’) (Figure 6A). This indicates a strong tendency toward leaf accumulation of TIAs across most cultivars.

When focusing on the concentration of specific anticancer alkaloids (sum of VLB and VCR), no significant differences were observed among cultivars at the leaf level, where values ranged from 13.5 µg·g^−1^ FW (‘C-BG’) to 31.7 µg·g^−1^ FW (‘C-Red’) (Figure 6B). However, in floral tissues, a contrasting pattern emerged, with significant variation across cultivars. ‘C-XDR-WT’ and ‘C-XDR-PN’ exhibited the highest concentrations, while ‘S-APR’ displayed the lowest (Figure 6B). Unlike what was described for the total anticancer-related TIAs (Figure 6A), only ‘C-Red’ and ‘C-XDR-WT’ showed significant organ-specific differences in VLB + VCR levels, ‘C-Red’ had 194% higher concentration in leaves, whereas ‘C-XDR-WT’ accumulated 159% more in flowers. Notably, the levels of these two alkaloids were approximately 1000 times lower than total TIA concentrations, showing the challenge of producing VLB and VCR at commercially viable levels.

**Figure 7:**
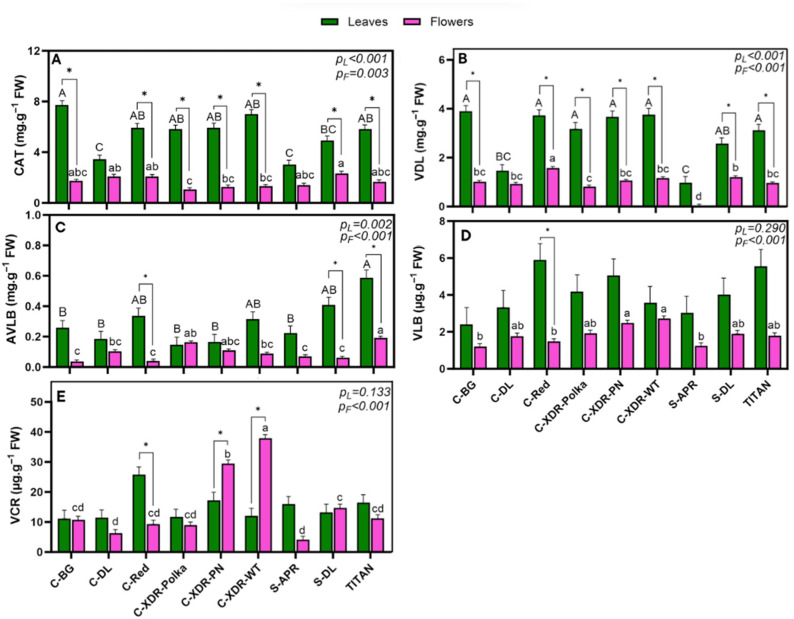
Concentration of each measured anticancer-related TIA [Catharanthine (CAT) (**A**), vindoline (VDL) (**B**), anhydrovinblastine (AVLB) (**C**), vinblastine (VLB) (**D**), and vincristine (VCR) (**E**)] per gram fresh weight (FW) in the leaves and in the flowers of nine *Catharanthus roseus* cultivars 35 days after transplanting to a vertical farming system. The cultivars tested were Cora Burgundy (C-BG), Cora Deep Lavender (C-DL), Cora Red (C-Red), Cora XDR Polka Dot (C-XDR-Polka), Cora XDR Punch (C-XDR-PN), Cora XDR White (C-XDR-WT), Sunstorm Apricot (S-APR) Sunstorm Deep Lilac (S-DL), and Titan Polka Dot (TITAN). Mean values are based on three independent replicates (n = 3), each composed of five plants. Error bars represent the standard error of the mean (SEM), calculated from the common variance. *p*-values indicate the *f*-probability for cultivar differences. Different lowercase and uppercase letters above the bars indicate statistically significant differences between cultivars within each organ – flowers and leaves, respectively – as determined by Tukey’s HSD test (*p* = 0.05). Additionally, a paired *t*-test (df = 2, critical t = 4.303) was performed to assess significant differences between leaves and flowers within each cultivar, with significant differences marked by an asterisk (*).

Among the five individual alkaloids quantified, CAT was the most abundant, ranging from 3.01 mg·g^−1^ FW (‘S-APR’) to 7.72 mg·g^−1^ FW (‘C-BG’) in leaves (Figure 7A), followed by VDL, which reached up to 3.90 mg·g^−1^ FW in ‘C-BG’ leaves (Figure 7B). In contrast, AVLB was synthesized at 10 times lower levels than VDL, with the highest concentration observed in ‘TITAN’ leaves (Figure 7C). Finally, the anticancer alkaloids VLB and VCR were detected in lower amounts, with ‘C-XDR-WT’ exhibiting the highest VCR concentration in flowers (Figure 7E). VLB was the least abundant alkaloid in this study, with concentrations ranging from 2.40 µg·g^−1^ FW (‘C-BG’) to 5.90 µg·g^−1^ FW (‘C-Red’) in leaves and from 1.23 µg·g^−1^ FW (‘S-APR’) to 2.72 µg·g^−1^ FW (‘C-XDR-WT’) in flowers (Figure 7D).

In our comparative analysis among cultivars, ‘C-DL’ and ‘S-APR’ consistently exhibited lower concentrations of the upstream alkaloids CAT and VDL in leaves (Figure 7A,B) and were the only cultivars with no significant differences in these alkaloids between leaves and flowers. In contrast, all other cultivars showed significantly higher CAT and VDL concentrations in leaves than in flowers. The most pronounced difference was observed in ‘C-BG’, with CAT and VDL levels 4.43 and 3.90 times higher in leaves, respectively (Figure 7A,B). For AVLB, ‘TITAN’ exhibited the highest concentration across both organs, reaching 0.59 mg·g^−1^ FW in leaves and 0.19 mg·g^−1^ FW in flowers (Figure 7C). In leaves, ‘TITAN’ was followed closely by ‘S-DL’, ‘C-Red’, and ‘C-XDR-WT’, which showed no significant differences among them. In flowers, AVLB was next highest in ‘C-XDR-Polka’ and ‘C-XDR-PN’. Only ‘C-Red’, ‘S-DL’, and ‘TITAN’ showed a significant difference in AVLB concentrations between organs, with AVLB levels in leaves being approximately 4.4 times higher (Figure 7C).

Although no significant differences in VLB concentration were detected among cultivars in leaves, ‘C-XDR-WT’ and ‘C-XDR-PN’ synthesized significantly higher VLB levels in flowers compared to ‘C-BG’, ‘C-Red’, and ‘S-APR’ (Figure 7D). A similar trend was observed for VCR: while leaf concentrations did not differ significantly among cultivars, both ‘C-XDR-WT’ and ‘C-XDR-PN’ showed significantly higher VCR accumulation in flowers, with 2.3-fold greater concentrations than in leaves. These two cultivars also exhibited the highest overall VCR concentrations, followed by ‘C-Red’, the only cultivar in which VCR levels were significantly higher in leaves than in flowers (Figure 7E).

**Figure 8:**
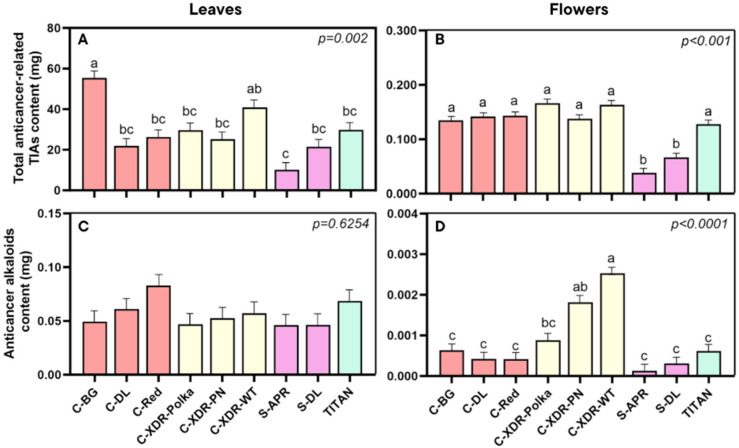
Total anticancer-related TIA content [measurements included catharanthine (CAT), vindoline (VDL), anhydrovinblastine (AVLB), vinblastine (VLB), and vincristine (VCR)] (**A**,**B**), along with the content of specific anticancer alkaloids (vinblastine and vincristine) (**C**,**D**) in the leaves (**A**,**C**) and in the flowers (**B**,**D**) of nine *Catharanthus roseus* cultivars 35 days after transplanting to a vertical farming system. The cultivars tested were Cora Burgundy (C-BG), Cora Deep Lavender (C-DL), Cora Red (C-Red), Cora XDR Polka Dot (C-XDR-Polka), Cora XDR Punch (C-XDR-PN), Cora XDR White (C-XDR-WT), Sunstorm Apricot (S-APR) Sunstorm Deep Lilac (S-DL), and Titan Polka Dot (TITAN). These values represent the alkaloid yield (calculated by multiplying the total organ dry weight × concentration) obtained in each cultivar and type of organ. Mean values are based on three independent replicates (n = 3), each composed of five plants. Error bars represent SEM, which is the mean of the standard errors based on the common variance. *p*-values indicate the f-probability for cultivar differences. Different letters above the bars indicate statistically significant differences between cultivars, as determined by Tukey’s HSD test at *p* = 0.05.

When analyzing alkaloid content (calculated as the product of concentration and organ biomass), leaves consistently yielded significantly higher amounts of TIAs than flowers: approximately 100 times more for both total anticancer-related TIAs and specific anticancer alkaloids (Figure 8). For instance, in terms of the total yield of anticancer-related TIAs in leaves (Figure 8A), ‘C-BG’ produced the highest amount, while ‘S-APR’ had the lowest, highlighting a marked cultivar effect. A similar trend was observed in flowers, where the series *Sunstorm* accumulated the lowest total alkaloid content, about 65% less than the other cultivars (Figure 8A). When examining the specific anticancer alkaloids (VLB and VCR), no significant differences in yield were found among cultivars in leaves (Figure 8C). In contrast, flower-derived yields of these alkaloids did vary significantly: ‘C-XDR-WT’ exhibited the highest content, followed by ‘C-XDR-PN’, both showing 4.2 times higher contents than most other cultivars. As noted earlier, flower yields remained, on average, 100 times lower than those obtained from leaves.

## 3. Discussion

### 3.1. Morpho-Physiological Diversity in Catharanthus roseus Cultivars

This study showed a large intraspecific morphological and physiological variability within the nine studied *C. roseus* cultivars, grown under VF conditions (Figure 2, Figure 3, Figure 4 and Figure 5). This variability was not only found when comparing series but also within the same *C. roseus* series, reinforcing the existence of cultivar-specific morpho-physiological adaptations to controlled VF conditions. For example, ‘C-XDR-Polka’ exhibited the highest total dry weight, while ‘S-DL’, ‘S-APR’, and ‘Titan’ showed substantially lower values.

Interestingly, large differences in photosynthetic rates did not always correlate with biomass accumulation. Within the *Sunstorm* series, ‘S-APR’ showed a 69% higher photosynthetic rate than ‘S-DL’, yet produced lower biomass than high-performing cultivars such as ‘C-XDR-Polka’. This is likely due to its relatively smaller LA, which constrained light capture and overall assimilate production. Conversely, cultivars such as ‘C-BG’, ‘C-XDR-WT’, and ‘C-XDR-Polka’ achieved greater biomass despite lower photosynthetic rates, likely benefiting from larger LA and possibly more efficient carbon partitioning strategies. This apparent disparity between photosynthetic efficiency and biomass has also been reported in other species, where LA—and thus light interception—was found to be a stronger determinant of growth than photosynthetic rate alone [33,34,35]. Furthermore, Leister (2023) [36] emphasized that the conversion efficiency of light into biomass varies among species and has not been a major focus in past agricultural improvement efforts. In addition, transpiration—closely tied to both stomatal conductance and LA—may also contribute to the observed variation in photosynthetic capacity. Cultivars with higher LA and conductance may experience greater gas exchange and evaporative cooling, potentially supporting higher CO_2_ uptake but also increasing water demand. Future studies incorporating transpiration rates and water-use efficiency metrics could provide deeper insights into these physiological trade-offs and help optimize cultivar performance under VF conditions.

In contrast, plant height was relatively uniform across cultivars, with only significant differences observed between ‘C-XDR-Polka’ and ‘S-APR’. The overall short stature of approximately 8.00 cm at harvest is ideal for vertical farming systems [37], making these cultivars well-suited for optimizing productivity in space-constrained environments.

### 3.2. Variability in Upstream Alkaloid Concentration and Potential Bottlenecks in Anticancer Biosynthesis

The cultivar significantly influenced the concentration of upstream alkaloids (CAT and VDL) in both leaves and flowers. ‘C-DL’ and ‘S-APR’ had notably lower CAT and VDL concentrations in leaves and were the only cultivars without significant differences between organs. In all others, leaves were strongly dominant. On average, CAT levels were 2.2 times higher than VDL, aligning with previous studies reporting CAT as the more abundant precursor [14,38,39], possibly due to greater biosynthetic activity or stability. However, despite the variability observed in upstream precursors, the limited accumulation of downstream anticancer alkaloids—particularly VLB—suggests that bottlenecks may lie beyond AVLB, in later conversion steps. This supports the hypothesis that AVLB is not the primary limiting factor, contrary to what was earlier proposed [15], and shifts attention toward the poorly characterized enzymatic conversion of AVLB into VLB. These patterns likely reflect genotype-dependent regulation of specific pathway steps, which may differently affect the final stages of anticancer alkaloid biosynthesis. Identifying cultivars with consistently high CAT and VDL levels, along with favorable conversion to downstream enzymes, will be key to optimizing selection for anticancer alkaloid production.

Previous research has reported trace or undetectable levels of AVLB in certain *C. roseus* tissues, suggesting it could represent a major biosynthetic bottleneck [15]. In our study, AVLB was detectable in both leaves and flowers, with significantly higher concentrations in leaf tissues for three of the nine cultivars. This cultivar-dependent difference supports previous suggestions that dimerization efficiency (CAT + VDL → AVLB) varies with genotype [22,23,24]. To assess downstream conversion efficiency, we calculated two key ratios: AVLB biosynthesis efficiency (based on CAT and VDL availability) ranged from 1.7% to 6.0%, while AVLB-to-VLB conversion efficiency ranged from only 0.9% to 4.2%. These findings suggest that, contrary to previous assumptions [15], the major bottleneck may lie beyond AVLB, and likely at the poorly characterized step converting AVLB to VLB. The observed low conversion rates across cultivars further support this hypothesis and reinforce the importance of evaluating not only precursor abundance but also enzymatic efficiency when selecting high-performing genotypes.

While VLB and VCR were generally found at lower concentrations than their precursors—especially VLB, which remained the least abundant alkaloid in all cultivars—notable cultivar-specific differences emerged at the floral level. In particular, ‘C-XDR-WT’ and ‘C-XDR-PN’ exhibited significantly higher VCR accumulation in flowers, whereas ‘C-Red’ synthesized more VCR in leaves. This organ-specific variation suggests cultivar-dependent differences in either alkaloid transport, storage, or tissue permeability. Although VLB and VCR biosynthesis is known to occur mainly in leaves [15,20,40], their presence in floral tissues, particularly in selected genotypes, likely results from post-synthetic translocation rather than biosynthesis in the floral tissues. This interpretation is consistent with the current literature indicating that the enzymatic reactions downstream of AVLB predominantly occur in vegetative organs [20,40].

VLB was consistently found at lower concentrations than VCR, confirming trends reported in previous studies [27,28,41]. This discrepancy may be attributed to the conversion of VLB into VCR, a biochemical step that remains poorly characterized [17], and whose efficiency likely influences the relative abundance of each compound in plant tissues.

Altogether, these results point to a complex, genotype-specific regulation of alkaloid partitioning. The high VCR levels in flowers of certain cultivars could reflect differences in intra-plant alkaloid trafficking mechanisms, such as long-distance transport, sink–source relationships, or tissue-specific permeability, that are not fully understood yet

### 3.3. Higher Alkaloid Content from Leaves than from Flowers

Our findings confirm that between leaves and flowers, leaves are the predominant organ for the production and accumulation of total anticancer-related TIAs, aligning with previous reports [15,21,28]. The total yield of TIAs from leaves was approximately 100 times higher than that from flowers, reinforcing their value as the primary target for commercial alkaloid extraction [16,21,42]. However, our study provides a novel perspective on cultivar-dependent variation in TIA partitioning; this distinction becomes minimal when considering actual yield: the content of VLB and VCR in flowers was on average 98.4% lower than in leaves (Figure 8C,D). Although some cultivars exhibited higher concentrations of VCR in flowers, the limited floral biomass drastically reduces their overall contribution to alkaloid yield. This highlights the practical importance of maximizing leaf production and leaf-targeted biosynthesis in cultivar selection and VF strategies aimed at optimizing the output of these important pharmaceutical compounds.

Although not directly addressed in this study, previous research has shown that alkaloid accumulation in *C. roseus* may decline with leaf age, likely due to metabolic downregulation or the onset of senescence [42]. This evidence supports the rationale behind our decision to harvest plants at 63 days after sowing, during a stage when leaves are still metabolically active and TIA biosynthesis is likely to be near peak levels. Optimizing the timing of harvest is particularly important in VF systems, where short production cycles and high-value compound yield are key to economic sustainability. Future studies should explore how different harvest stages influence the accumulation of dimeric alkaloids and antioxidants to further refine crop planning. Additionally, research could assess the potential benefits of extending the VF phase to earlier developmental stages, rather than using it exclusively for the second half of the growth cycle. This could help induce earlier biomass accumulation and specialized metabolite production, potentially shortening the ideal crop cycle for anticancer alkaloid harvest.

### 3.4. Comparative Analysis of the Alkaloid Concentration with Literature Values

The comparative analysis, performed in this section, focuses on leaf alkaloid concentration of *C. roseus* reported in previous studies, with the values achieved in the current research. A direct comparison of the flower-derived alkaloids was not possible due to the lack of quantitative data on floral tissues. This analysis clearly shows that the measured concentrations of TIAs at leaf level outperformed all the values achieved in the analyzed literature (Table 1). Specifically, VCR and VDL concentrations in the present research were 8.73 and 5.85 times higher than those previously reported, respectively [14,30,38,39,41,42,43,44,45], suggesting that VF conditions boost their production when compared with conventional production systems.

For AVLB, our study found concentrations 3.46 times higher than those reported by Carqueijeiro (2013) [38]. Given that AVLB synthesis requires a 1:1 ratio of CAT and VDL [18], the observed higher content of CAT in ‘C-BG’, ‘C-Red’, and the ‘Cora-XDR’ series suggests that VDL availability may be the primary limiting factor in AVLB production. This indicates that optimizing AVLB yields may require strategies to enhance VDL content rather than solely focusing on the peroxidase-mediated coupling reaction. These findings align with previous studies [15,27], which have suggested that precursor availability can significantly impact alkaloid biosynthetic flux.

In contrast, VLB levels remained relatively consistent with those reported by Fukuyama research group (2013, 2017, 2023) [39,43,45], suggesting a regulatory threshold for its accumulation. However, VCR displayed a more pronounced increase, reaching levels 8.73 times higher than those documented by Levingston (1983) [41]. Given that plants favor oxidative metabolism, it is plausible that VCR tends to accumulate at higher levels than VLB [28]. This further supports the notion that the rate-limiting step in the pathway may not be the VLB-to-VCR conversion, but rather the AVLB-to-VLB transformation. The latter remains poorly characterized, as the enzyme or enzymes responsible for this conversion are yet to be identified [15].

Altogether, these findings show the potential of VF systems to enhance alkaloid biosynthesis through optimized environmental parameters that regulate specialized metabolite production. From an industrial perspective, previous estimates suggest that 500 kg of dried leaf biomass is required to extract 1 g of VLB, whereas about 530 kg of plant material is needed to obtain 1 g of VCR [27,28,41]. Here, we demonstrate that under the controlled VF conditions used in this study, the cultivars with the highest VCR concentration (namely ‘C-Red’) can significantly increase alkaloid production, reducing the biomass required for commercial extraction to 38.8 kg of fresh leaves to obtain 1 g VCR and 169.5 kg to harvest 1 g VLB.

### 3.5. Toward Molecular Farming of Catharanthus roseus Under Vertical Farming Systems

Previous large-scale screenings have evaluated up to 64 cultivars [14,30], but only quantified upstream alkaloids such as CAT and VDL. To our knowledge, our study is the first to extend this scope to the pharmacologically relevant bis-indole alkaloids VLB and VCR, providing novel insights into their cultivar-dependent production and partitioning between leaves and flowers under VF conditions. While our study focused on nine cultivars of *C. roseus*, they were strategically selected from diverse commercial series known for their contrasting growth traits, offering a representative, but not exhaustive, snapshot of intraspecific variability. In spite of this limited number of studied cultivars, it was clearly demonstrated that a high intraspecific variability exists for the studied traits, including for the anticancer alkaloid biosynthesis and partitioning. Moreover, our findings show that exposing *C. roseus* to VF conditions during just the final 35 days was sufficient to stimulate a positive alkaloid biosynthetic response. This demonstrates the high adaptability of *C. roseus* to VF systems. Optimizing the growth stage for alkaloid extraction is also essential, since older *C. roseus* leaves tend to accumulate lower levels of bioactive alkaloids, likely due to metabolic downregulation or leaf senescence [42]. This further emphasizes the importance of precisely managing timing and growth stage under VF conditions to maximize pharmaceutical yields. Future studies should explore different cultivation strategies and environmental settings within VF systems, not only to optimize the balance between biomass and alkaloid production, but also to assess alkaloid accumulation dynamics throughout the entire crop cycle. Monitoring these dynamics over time could help identify the biosynthetic peak and guide more precise scheduling of elicitation treatments, ultimately improving pharmaceutical yield efficiency in short-cycle vertical molecular farming.

Recent advancements in digital agriculture, including the integration of deep learning and morphological algorithms, may further optimize the screening of plant-derived compound production in VF systems. For instance, Wang et al. (2024) [46] demonstrated the effectiveness of the Litchi-YOSO model in localizing plant structures with high precision, supporting scaled, automated cultivation and harvesting. Applying such approaches to *C. roseus* cultivation could enhance phenotyping accuracy, alkaloid yield prediction, and harvesting efficiency, paving the way for precision molecular farming.

Finally, additional studies are needed to investigate genotype-dependent regulatory mechanisms, organ-specific transport dynamics, and the strategic use of elicitors—such as UV light—to further enhance alkaloid output without compromising vegetative growth. These approaches could contribute to the refinement of vertical molecular farming systems aimed at high-value compound production.

## 4. Materials and Methods

### 4.1. Plant Material and Growing Conditions

Seedlings of nine *Catharanthus roseus* (L.) cultivars (Syngenta Flowers) were acquired from a commercial nursery (Group Roig—Viveros Pereira; Valencia, Spain) four weeks after sowing. These cultivars were selected from four series, chosen for their growth performance and broad disease resistance: ‘Cora’ series [Cora Deep Lavender (‘C-DL’), Cora Red (‘C-Red’), Cora Burgundy (‘C-BG’)]; ‘Cora XDR’ series [Cora XDR White (‘C-XDR-WT’), Cora XDR Polka Dot (‘C-XDR-Polka’), Cora XDR Punch (‘C-XDR-PN’)], ‘Sunstorm’ series [Sunstorm Deep Lilac (‘S-DL’), Sunstorm Apricot (‘S-APR’)], and Titan Polka Dot (‘TITAN’). At transplanting date, seedlings were selected for uniformity and the initial total dry weight (0.074 ± 0.022 g), number of leaves (4.7 ± 0.5) and plant height (4.9 ± 0.6 cm) were recorded in 10 rooted seedlings per cultivar within that uniform batch. No significant differences were observed among cultivars at this stage. Seedlings were transplanted into 8 cm diameter plastic pots containing a growing medium comprising 50% humus, 17% peat (0–40 mm), 17% perlite, and 15% coco peat, enriched with a balanced fertilizer (16N-11P-10K + 2Mg + micronutrients). Plants were cultivated in controlled climate chambers maintained at 24.0 ± 0.8 °C and 80% relative humidity at crop level (LogTag^®^ TRIX-8, Auckland, New Zealand). The light regime consisted of a photoperiod of 12 h light supplied with a dichromatic spectrum of red (667 nm) and blue (450 nm) LEDs at a 2:1 ratio, reaching a photosynthetic photon flux density (PPFD) of 150 μmol·m^−2^·s^−1^ (spectrophotometer Optimum SRI-2000; Hsinchu, Taiwan).

### 4.2. Morphological and Physiological Measurements

At the end of the experiment, several morphological parameters were recorded, including organ fresh weight, plant height, and total LA (LI-3100C Leaf Area Meter, LI-COR, Lincoln, NE, USA). Roots, stems, leaves, and flowers were dried at 90 °C for 72 h in a ventilated oven, and the total plant dry weight was the sum of all these dried organs. Specific leaf area (SLA) was calculated as the ratio of LA to LDW (cm^2^·g^−1^ DW). Root/shoot ratio was also calculated on a dry weight basis (g·g^−1^ DW).

Physiological measurements included the chlorophyll content measured on the youngest fully expanded leaf using the non-destructive chlorophyll meter SPAD (Konica Minolta SPAD-502 model, Minolta, Tokyo, Japan). Photosynthetic performance was also assessed, using an Infrared Gas Analyzer (IRGA LI-6400XT, LI-COR Inc., Lincoln, NE, USA). Parameters measured included the net photosynthesis rate (μmol CO_2_·m^−2^·s^−1^) and stomatal conductance (μmol H_2_O·m^−2^·s^−1^).

### 4.3. Alkaloid Extraction and LC-MS Methods

Alkaloid extraction followed the protocol described by Carqueijeiro (2016) [47]. Fresh *C. roseus* leaves were flash-frozen in liquid nitrogen (−70 °C) and ground into a fine powder using a mortar and pestle. Approximately 150 mg of the powdered sample was transferred into a 2 mL Eppendorf tube, and 1 mL of methanol (MeOH) was added. The sample was vortexed for 2 min, followed by sonication for 30 min. Thereafter, the microtubes were centrifuged at 12,000 rpm for 10 min, and the supernatant was collected and filtered through a 0.45 μm membrane filter before injection into the liquid chromatography–mass spectrometer. The samples were analyzed by LC-ESI-SIM in a Finningan Surveyor Plus HPLC system (Thermo Scientific, Waltham, MA, USA) coupled to Finningan LCQ Deca XP Plus mass detector with an electrospray ionization source (ESI) operating at room temperature and a quadrupole ion trap. The system was controlled, and the information recorded by the Thermo Xcalibur™ Qual Browser software version 2.2 SP1.48 (Thermo Fisher Scientific, Waltham, MA, USA). The separation was carried out under the same conditions described by Jeong and Lim (2018) [48] with some modifications. The column and the solvents are the same, but the contents of mobile phase were varied from 35% and not 40% of B from 5 min to 10 min. The mass spectra were acquired in positive mode in the range between 300 *m*/*z* and 1000 *m*/*z* from ESI source with the conditions set to capillary temperature of 325 °C, capillary exit offset of 5 KV, capillary voltage of 15 V, and tube lens of 50 V. Nitrogen was used as nebulizing and drying gas at 40 and 15 (arbitrary units) respectively.

The LC-MS output chromatograms were analyzed using XCalibur™ software version 2.2, with each alkaloid identified by its observed ionic mass (*m*/*z*): catharanthine (CAT: 337), vindoline (VDL: 457), vincristine (VCR: 825), anhydrovinblastine (AVLB: 793), and vinblastine (VLB: 811). The alkaloid concentrations in the samples were quantified in mg·g^−1^ fresh weight (FW) using calibration curves based on five known concentrations of each standard alkaloid. Alkaloid yield was calculated using the following equation:

$$Alkaloid\;Yield\;\left(mg\right)=Alkaloid\;Concentration\;\left(mg\cdot g^{-1}\right)\times \;Biomass\;(g)$$

### 4.4. Experimental Design and Statistical Analysis

The experiment ended 35 days after transplanting the rooted seedlings and placing them in the VF system. This represented a total cultivation period of 63 days (from sowing to harvest), and it corresponded to the full-bloom stage of development. This timing was selected due to its biological relevance and to ensure enough flower biomass for alkaloids’ quantification. Moreover, previous studies have shown that alkaloid concentration may decline in older tissues [42,49]; thus, prolonging the trial could lead to lower levels. A similar cultivation period was used in other experiments, where the plants were analyzed following 63 to 70 days of cultivation [43,50,51].

The experiment followed a complete randomized design and was conducted three times, representing three independent replicates (each composed of five plants per cultivar). To minimize border effects, plants were randomly repositioned every three days. For the analysis of morphological and physiological parameters, the dataset comprised 27 averaged measurements (9 cultivars × 3 independent replicates), representing a total of 135 plant samples (5 plants per cultivar per replicate). Differences among cultivars were assessed using one-way ANOVA, followed by Tukey’s post hoc test for pairwise comparisons at a 95% confidence level (*p* = 0.05). For alkaloid quantification, conducted on leaves and flowers, the dataset consisted of 54 measurements (9 cultivars × 2 organs × 3 replicates). Pooled samples of five plants per replicate (n = 3) per treatment were used for analysis. To evaluate differences between leaves and flowers within each cultivar, a paired *t*-test was performed (df = 2, critical *t* = 4.303). All statistical analyses were conducted using IBM SPSS Statistics version 29.0.2, and graphs were generated using GraphPad Prism version 10.1.0.

## 5. Conclusions

This study demonstrates that VF enhances the overall biosynthesis of key anticancer alkaloids—mainly VCR—in *Catharanthus roseus*, outperforming yields reported in previous studies and reinforcing VF’s potential as a scalable and sustainable platform for PMF. Cultivar selection emerged as a critical factor influencing not only the total production of these compounds but also their organ-specific distribution, with notable differences in accumulation patterns across leaves and flowers. For example, cultivars such as ‘C-XDR-WT’ and ‘C-XDR-PN’ exhibited unexpectedly high VCR levels in floral tissues, highlighting genotype-specific differences in biosynthesis and partitioning.

Among the studied compounds, VCR proved to be a reliable marker for identifying cultivars with high anticancer alkaloid yield, offering a more targeted selection criterion than total biomass or precursor content alone. Across all cultivars, leaves consistently yielded higher amounts of alkaloids compared to flowers, confirming their role as the primary site of TIA accumulation.

Importantly, our findings show that a 35-day VF growth phase was sufficient to produce relevant alkaloid levels, supporting the feasibility of efficient, multi-cycle, year-round production. Furthermore, the observed bottleneck in the biosynthetic pathway appears to lie downstream of AVLB, underscoring the importance of improving late-stage conversion efficiency to optimize the output of these pharmaceutical compounds. Altogether, these insights support the integration of VF with targeted cultivar selection strategies to enhance the productivity and efficiency of high-value alkaloid production systems.

## Figures and Tables

**Table 1:** Summary of leaf alkaloid concentrations in *Catharanthus roseus* reported in previous studies, expressed on dry weight (DW) and/or fresh weight (FW) basis, alongside the corresponding values measured in the present study. References indicate sources of literature-reported values. Abbreviations: CAT—catharanthine, VDL—vindoline, AVLB—anhydrovinblastine, VLB—vinblastine, VCR—vincristine.

Measured Terpenoid Indole Alkaloids	Literature Data		References	Present Study	
	DW Basis	FW Basis		Measured Concentrations	Fold Increase ^x^
CAT (mg·g^−1^)	0.08–6.00	0.40–6.50	[14,30,38,39,41]	3.01–7.72 mg·g^−1^ FW	1.55
VDL (mg·g^−1^)	2.08–7.00	0.11–1.40	[14,30,38,39,43]	0.98–3.90 mg·g^−1^ FW	5.85
AVLB (mg·g^−1^)	------	0.07	[38]	0.14–0.59 mg·g^−1^ FW	3.46
VLB (µg·g^−1^)	5.00–80.0	------	[39,45]	23.3–57.3 µg·g^−1^ DW	0.95
VCR (µg·g^−1^)	------	1.90	[41]	11.1–25.8 µg·g^−1^ FW	8.73

^x^ Calculated based on the average value of the displayed range of the literature data and of the measured concentrations.

## Data Availability

The data are contained within the article.

## Abbreviations

The following abbreviations are used in this manuscript:

AVLB	Anhydrovinblastine
CAT	Catharanthine
C-BG	Cora Burgundy
C-DL	Cora Deep Lavender
C-Red	Cora Red
C-XDR-Polka	Cora XDR Polka Dot
C-XDR-PN	Cora XDR Punch
C-XDR-WT	Cora XDR White
DAT	Deacetylvindoline 4-O-Acetyltransferase
D4H	Deacetoxyvindoline 4-Hydroxylase
ESI	Electrospray Ionization Source
LA	Leaf Area
PMF	Plant Molecular Farming
PPFD	Photosynthetic Photon Flux Density
PRX1	Peroxidase 1
S-APR	Sunstorm Apricot
S-DL	Sunstorm Deep Lilac
SGD	Strictosidine β-Glucosidase
SLA	Specific Leaf Area
STR	Strictosidine Synthase
TDW	Total Dry Weight
TFW	Total Fresh Weight
TIA	Terpenoid Indole Alkaloid
TITAN	Titan Polka Dot
VDL	Vindoline
VCR	Vincristine
VF	Vertical Farming
VLB	Vinblastine
